# Local/Topical Antibiotics for Peri-Implantitis Treatment: A Systematic Review

**DOI:** 10.3390/antibiotics10111298

**Published:** 2021-10-25

**Authors:** Pier Carmine Passarelli, Andrea Netti, Michele Antonio Lopez, Eleonora Favetti Giaquinto, Giuseppe De Rosa, Gianmarco Aureli, Alina Bodnarenko, Piero Papi, Anna Starzyńska, Giorgio Pompa, Antonio D’Addona

**Affiliations:** 1 Department of Head and Neck and Sensory Organs, Division of Oral Surgery and Implantology, Institute of Clinical Dentistry, Gemelli Foundation for the University Policlinic, Catholic University of the “Sacred Heart”, 00168 Rome, Italy; piercarmine.passarelli@unicatt.it (P.C.P.); Eleonora.favetti@gmail.com (E.F.G.); giuseppederosa1410@gmail.com (G.D.R.); gianmarco.aureli@gmail.com (G.A.); Antonio.daddona@policlinicogemelli.it (A.D.); 2 Unit of Otolaryngology, University Campus Bio-Medico, 00128 Rome, Italy; micheleantonio.lopez@gmail.com; 3 Department of Oral Surgery, Medical University of Gdańsk, 7 Dębinki Street, 80-211 Gdańsk, Poland; a.bodnarenko@gumed.edu.pl (A.B.); anna.starzynska@gumed.edu.pl (A.S.); 4 Department of Oral and Maxillofacial Sciences, Policlinico Umberto I, “Sapienza” University of Rome, 00161 Rome, Italy; Piero.papi@uniroma1.it (P.P.); giorgio.pompa@uniroma1.it (G.P.)

**Keywords:** chemical adjuvants, peri-implantitis, peri-implant mucositis, local antibiotics, minocycline, clinical practice guidelines

## Abstract

Most studies indicate that the mechanical removal of the bacterial biofilm from the implant surface is the central goal of peri-implantitis therapy. However, controversial results in the treatment of peri-implantitis have led to the consideration of additional strategies that include surgical approaches and chemical adjuvants. Local/topical antibiotics, such as minocycline, azithromycin, tetracycline, amoxicillin, doxycycline, and metronidazole, may improve the efficacy of the definitive treatment of the disease, but the lack of conclusive findings prevents their use in clinical practice. This systematic review aimed to evaluate the effect of local/topical antibiotics for peri-implantitis treatment. Randomised controlled studies (RCT) on patients with peri-implantitis and comparing the efficacy of local/topical antibiotics vs. placebo or mechanical debridement were included. A systematic search strategy was carried out using three registered databases (PubMed, Web of Science, and Scopus). RoB2 was used to assess risk of bias. Five RCTs were identified (*n* = 250 patients and 333 implants). Contrast results emerged among the included studies, and a high heterogeneity level was observed. Risk of bias revealed some concerns for three studies out of five, while one study was judged at high risk. Only one study analysed the limitations of its findings. Overall, local antibiotic use can be considered a valid approach in the treatment of peri-implantitis. Therefore, future long-term clinical trials with standardised protocols and antibiotics with similar biological activity profiles should be tested to achieve a valid and definitive conclusion.

## 1. Introduction

Peri-implantitis and peri-implant mucositis are included within the peri-implant disease concept [1]. Peri-implantitis prevalence has been observed in 19.83% of subjects and 9.25% of implant sites, while 46.83% of subjects and 29.48% of implants develop peri-implant mucositis [2].

Peri-implantitis is a progressive and irreversible disease of implant-surrounding hard and soft tissues, associated with progressive bone loss, bone resorption, decreased osseointegration, increased pocket formation, and purulence [2,3]. Peri-implant mucositis refers to a reversible inflammatory process in the soft tissues and connective tissues adjacent to the implant [4].

Although smoking, poor plaque control, absence of keratinised mucosa, excess cement, diabetes, and other systemic conditions have been identified as risk factors related to peri-implantitis [5], dental plaque or biofilm are considered the principal etiological factors in periodontal disease [6]. Furthermore, analysing the aetiology, pathophysiology, risk assessment, and therapy of peri-implantitis, many studies have observed a strong level of similarity between the two pathologies and suggested similar therapeutic approaches [7,8].

To date, the etiological studies of peri-implant diseases are limited, especially if compared to periodontal diseases [4,8]. Many etiological factors [5,9,10] and causative organisms (e.g., *Prevotella intermedia*, *Tannerella forsytbia*, *Eikenella corrodens*, *Filifactor alocis*, and *Aggregatibacter actinomycetemcomitans*) [1,4,8,9,11] of periodontal disease may also be identified in the onset of peri-implantitis [12]. According to two recent studies, peri-implantitis is the result of the action of several different species of bacteria, including *Tannerella forsytbia* and *Porpbyromonas gingivalis* [2,5]. A systematic and meta-analysis review analysing the peri-implantitis lesions and associated bacteria and comparing the results with a control group of individuals with healthy implants demonstrated a higher prevalence of *Prevotella intermedia*, *Tannerella forsytbia*, and *Aggregatibacter actinomycetemcomitans* [11]. The histological data obtained from human biopsy specimens identified polymorphonuclear leukocytes, plasma cells, macrophages, and lymphocytes in the connective tissue around the implant with peri-implant inflammation [13].

Consequently, peri-implantitis highly depends on the imbalance of host–microbiome periodontal homeostasis as it occurs in periodontal disease, in which susceptible hosts facilitate dysbiosis and the development of plaque biofilm-associated microbial communities that consist of pathobionts and keystone pathogens [9]. Concomitantly with the host’s response, their synergistic virulence causes destructive inflammation. Through the facilitation of dysbiosis and bone loss through inflammation, systemic complications are observed together with tooth and implant loss [5,9,10]. Conversely, variations in the number of pathogen species populations within the plaque biofilm and oral microbiome trigger the host’s immunological reaction, facilitating periodontitis and peri-implantitis less often [14].

Most of the published peri-implantitis therapeutic strategies are founded on periodontitis treatment strategies: the colonisation of pathogens on the implant and dental surfaces has a common pathophysiology involving the microbial film, as in the periodontal disease [14]. Therefore, treatment in peri-implantitis aims to reduce the pathogen load by shifting the bacteria’s biofilm composition. The volume of plaque biofilm is reduced through mechanical instrumentation with air powder abrasive, metal curettes, non-metal curettes (carbon, plastic, resin-reinforced, and resin-un-reinforced), an ultrasonic scaler with a metal or plastic tip, and implantoplasty, which, by reducing the roughness of the implant surfaces, reduces plaque adherence [15]. In addition to having quantitative efficacy in the overall volume of the biofilm, this reduction represents, from an etiological point of view, a qualitative variation in the biofilm composition [16,17].

The therapeutic gold standard for peri-implantitis is dynamic, comprising mechanical instrumentation, regular periodontal supportive maintenance therapy, and home care. For the control of peri-implantitis, mechanical debridement alone has failed to thoroughly remove bacterial biofilms due to their location and accessibility; therefore, a single therapy can be unreliable in disease control [8,15]. Furthermore, mechanical debridement alone is insufficient for immunocompromised patients, especially those with altered immune capacity. The Cumulative Interceptive Supportive Therapy (CIST) protocol appears to be a viable alternative in the approach to peri-implant disease, depending on the severity grade [3], given that the reduction of peri-implant inflammation and ideally peri-implant tissue regeneration is the central therapeutic goal. CIST protocol is based on regular recalls of the implanted patient and repeated assessments of plaque, bleeding, suppuration, pockets, and radiological evidence of bone loss. Local/topical and systemic antibiotic administration represents a fundamental step to avoid or delay regenerative or resective surgery [18]. In 2004, it was modified and called the AKUT-concept, divided into four stages: stage A (pocket depth (PD) < 3 mm, plaque, and/or bleeding on probing (BOP)) involves mechanical cleaning, polishing, and oral hygiene instructions; stage B (PD 4–5 mm and no bone loss) involves adding antiseptic regimens (e.g., chlorhexidine) to the stage A treatment; stage C (PD > 5 mm, radiological bone loss (RBL) < 2 mm) involves adding microbiological testing and local/topical or systemic antibiotic therapy to the stage B treatment; and, finally, stage D (PD > 5 mm and RBL > 2 mm) involves regenerative or resective surgical treatment [19].

Therefore, adjunct methods involving chemical methods, such as hydrogen peroxide, tetracycline, saline-soaked cotton pellets, citric acid, and chlorhexidine, have been proven fundamental in the treatment of peri-implantitis [15], together with other adjuvant methods including phototherapy and laser therapy, such as a continuous carbon dioxide laser [20,21].

**Scheme 1:**
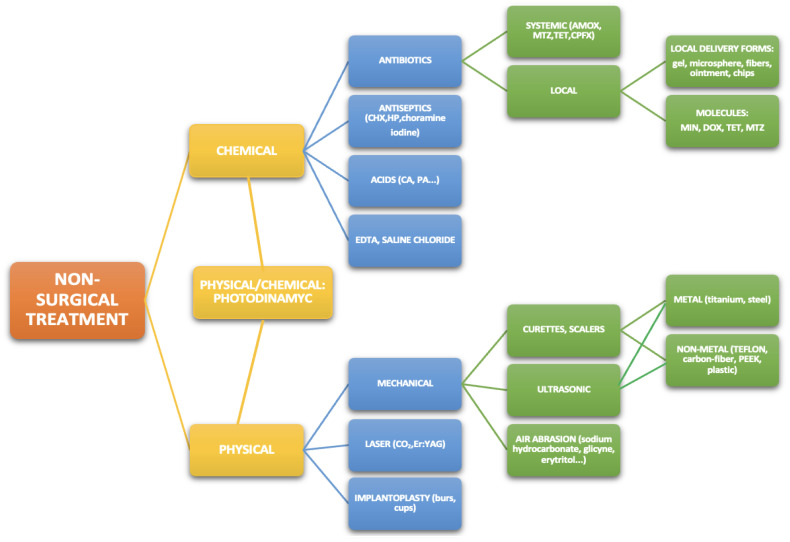
Non-surgical treatment methods of peri-Implantitis and peri-Implant mucositis. Legend: AMOX: amoxicillin, CA: citric acid, CHX: chlorhexidine, CPFX: ciprofloxacin, DOX: doxycycline, HP: hydrogen peroxide, MIN: minocycline, MTZ: metronidazole, PA: phosphoric acid, TET: tetracycline.

**Scheme 2:**
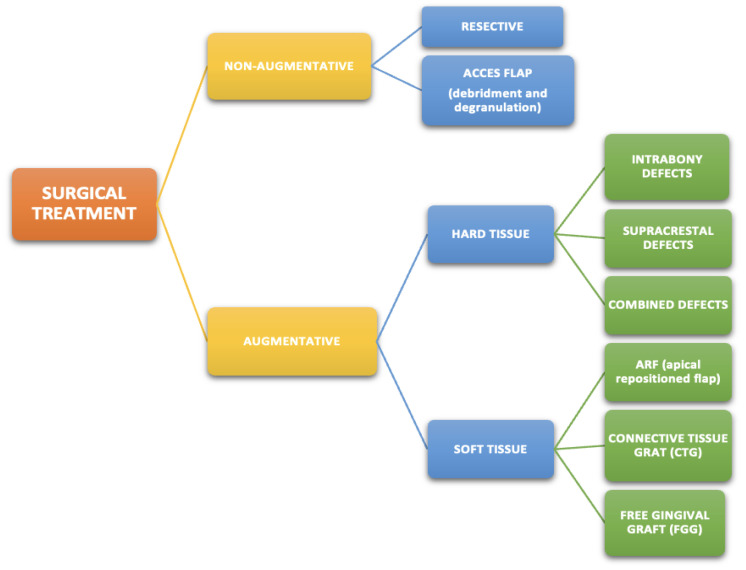
Surgical treatment methods of peri-implantitis.

The need for surgery decreases in peri-implantitis with anti-infective chemotherapy and mechanical debridement. A meta-analysis concluded that the effective management of peri-implantitis improves the retention of dentition throughout the lifetime [22]. Management of plaque biofilm in peri-implant mucositis makes it reversible. However, this approach could be revealed to be insufficient in more severe cases, and other nonsurgical procedures or surgery become treatments of choice [16] (Scheme 1 and Scheme 2).

Lan et al. introduced personalised alginate rings/poly-caprolactone loaded with metronidazole to ensure the constant release of the drug to maintain an appropriate concentration effective for the elimination of colonising bacterial biofilms. The study reported that the above method potentially minimises the development of a bacterial biofilm and, therefore, the therapy is applicable for the prevention of peri-implantitis [23].

In the treatment of peri-implantitis, it is becoming a standard practice to administer antibiotics and/or antiseptics locally to individuals with the disease, especially in moderate- to severe-progression PD [24]. To achieve a continuous release of antibiotics and sustain the required levels of antibiotics at the infected sites, controlled-release devices, including microcapsules, polymeric fibres, chips, and gels, have been proposed [25,26]. These kinds of devices allow the use of several antibiotics, such as metronidazole, minocycline, doxycycline, and tetracycline, and have been shown to ensure a steady, elevated antibiotic agent concentration in the periodontal pocket (gingival crevicular fluid) for an extended period before removal or antibiotic agent degradation [27]. For example, after 7–14 days, tetracycline fibre devices should be removed, whereas doxycycline polymer devices and minocycline microspheres require no removal since they are biodegradable [27,28].

Different forms of tetracycline for local delivery are available and have been tested on periodontitis, such as nano-particles [29], fibres [30,31,32], and ointment [29]. The use of tetracycline fibres as an adjunct to scaling and root planning resulted in periodontal health improvement and, therefore, is a reliable treatment [33]. The combination of scaling and root planning with tetracycline fibres is a more effective therapeutic approach than scaling and root planning alone when clinical parameters such as clinical attachment level (CAL) and PD are considered [31].

In the clinical context, local/topical antibiotic adjuncts in the mechanical treatment of peri-implantitis have always been applied [15] without being supported by firm recommendations and guidelines. Therefore, this review aimed to evaluate the effect of local antibiotics on peri-implantitis in reducing peri-implantitis signs.

## 2. Methods

The materials and methods were based on the PRISMA (Preferred Reporting Items for Systematic Reviews and Meta-Analysis) guidelines [34]. A search strategy was carried out on PubMed, Embase, and Web of Science from January to September 2021, without time and language restrictions.

The components of the PICOS question were as follows: (Patients) patients with periimplantitis; (Intervention) local antibiotics, topical antibiotics, or antibacterial agents; (Comparison) mechanical debridement alone or placebo procedure; (Outcome) clinical and microbiological outcome; (Study Design) RCT.

The keywords used in this search were: (peri-implantitis) AND (antibiotics OR local antibiotics OR topical antibiotics). Grey literature was searched using the Open Grey database (www.opengrey.eu, accessed on 13 September 2021) with the same search strategy used for the other databases. Additionally, several journals were hand-searched manually.

The first (title/abstract screening) and second (full-text assessment) steps of the search process were performed by two independent reviewers (P.C.P. and A.N.), and any disagreement was discussed until a decision was made by consensus.

### 2.1. Study Selection

The complete list of articles obtained through the search was scrutinised to remove duplicates and select the potentially relevant articles based on the title to answer the research question. Subsequently, abstract screening was performed as well. The eligible studies were independently selected by two reviewers (P.C.P. and A.N.). From the remaining potentially relevant articles, those that met the inclusion and exclusion criteria were selected through full-text reading. The reasons for exclusion were recorded.

The subsequent article selection was independently made by two authors (P.C.P. and A.N.). When there was disagreement, a third experienced reviewer (M.A.L.) was consulted to achieve a consensus.

### 2.2. Inclusion/Exclusion Criteria

The inclusion criteria were as follows: (i) studies conducted on patients with peri-implantitis defined as bleeding and/or suppuration on probing, peri-implant PD ≥ 4 or 5 mm; (ii) studies comparing the efficacy of local/topical antibiotics vs. placebo or mechanical debridement; (iii) randomised controlled studies (single- or double-blinding).

### 2.3. Data Extraction

Two reviewers (P.C.P. and A.N.) independently extracted the data from the full texts of the studies that fulfilled the inclusion criteria. Disagreements were resolved through team discussions. The primary outcomes analysed in this review were BOP, probing pocket (PPD), PD. In addition, microbiological analysis, plaque index (PI), and gingival index (GI) were also reviewed as secondary outcomes.

Data extraction was organised in tables that included the following information:

- Study characteristics: name of the first author, year, country, disease (peri-implantitis or peri-implant mucositis), study design, blinding, type of intervention (surgical vs. non-surgical), follow-up;
- Participant characteristics: implant numbers/subjects, inclusion criteria;
- Treatment and control groups characteristics;
- Primary and secondary outcomes;
- Results

### 2.4. Risk of Bias

The quality of each RCT was independently assessed according to the Cochrane Risk of Bias Tool (RoB2) by two reviewers. Five domains of bias (i.e., randomisation process, deviations from intended interventions, missing outcome data, measurement of the outcome, and selection of the reported results) were evaluated and reported. The Cochrane Handbook for Systematic Reviews of Interventions [35] was used. A judgement of “high” indicated a high risk of bias, “low” indicated a low risk of bias, and “some concerns” indicated the presence of bias due to lack of information or uncertainty about the potential for bias. The studies were categorised as having a low or high risk of bias or some concerns. Any discrepancy in the assessment of RoB2 was discussed to attain a consensus.

## 3. Results

### 3.1. Study Selection

**Scheme 3:**
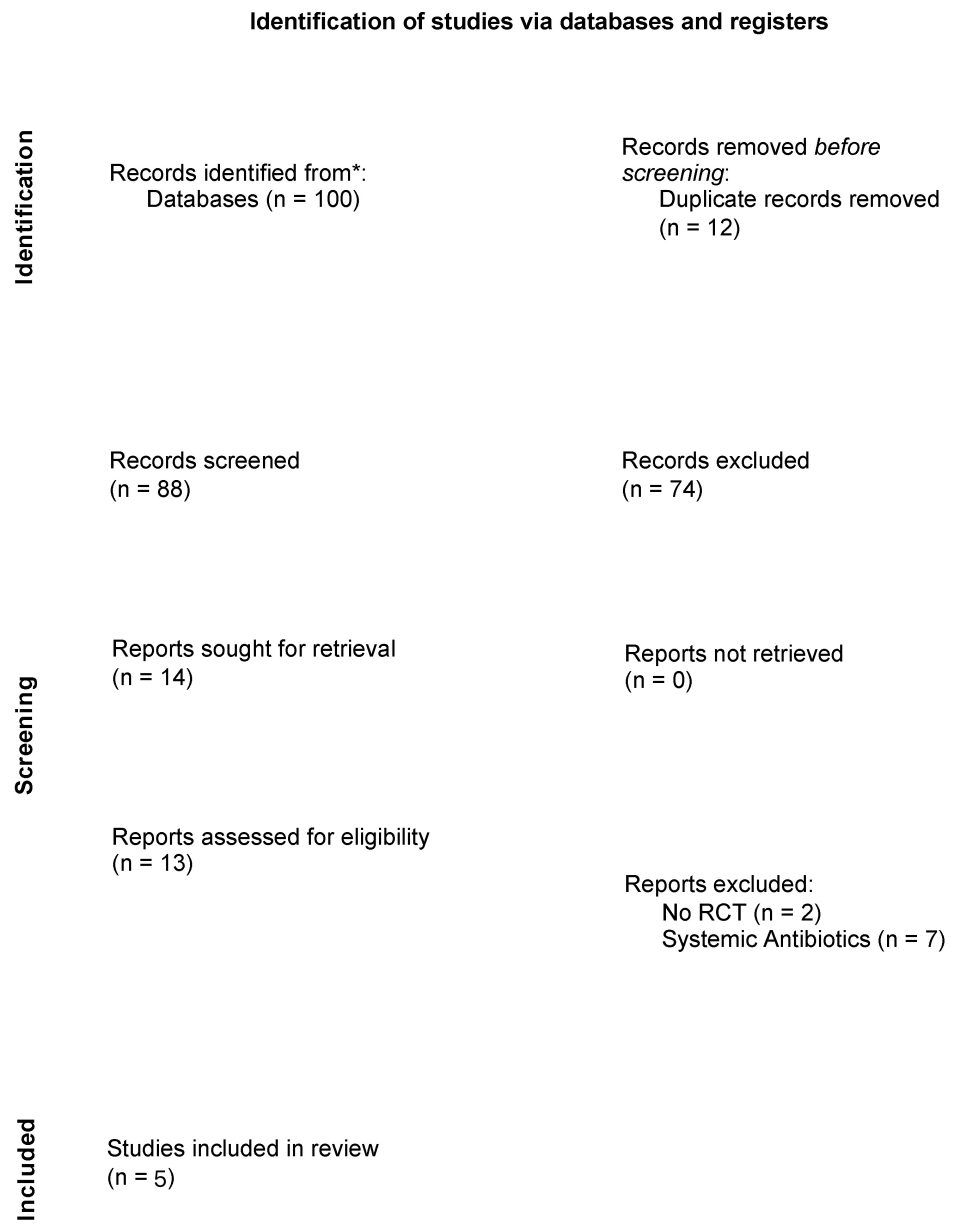
PRISMA 2020 flow diagram—Source: http://www.prisma-statement.org, accessed on 13 September 2021. * Total number of records across all databases was reported.

A flow diagram of the search strategy results is presented in Scheme 3. After the removal of 12 duplicates, a total of 88 articles were obtained. From these 88 articles, 74 studies were excluded after reading their titles and abstracts. Finally, 14 studies were selected for full-text reading. Of these 14 studies, two studies were not RCTs, and seven studies used systemic antibiotics. Therefore, a total of five studies were included in the review [28,36,37,38,39].

### 3.2. Risk of Bias

**Scheme 4:**
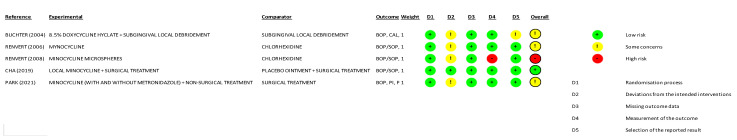
Risk of bias.

The overall risk of bias assessment of the included studies is presented in Scheme 4. We judged one study at low risk of bias [38]. Three studies were considered with some risk of bias because people delivering the intervention were aware of participants’ assigned intervention during the trial, and no appropriate analyses were carried out to estimate the effect of assignment to intervention [36,37,39]. Finally, one study [28] was judged at high risk of bias because, although the participants were randomised, more implants were allocated to the test treatment. 

### 3.3. Study Characteristics

We provide a descriptive summary of the information on participants, treatments, and comparisons in Table 1. We included a total of five RCTs: two carried out in Korea [37,38], two in Sweden [28,36], and one in Germany [39].

One study included more than 100 participants [37], while the remaining four included less than 50 patients [28,36,38,39]. The median follow-up was six months (IQR: 4–12 months). Four studies used minocycline local/topical antibiotics [28,36,37,38], while only one study used doxycycline [39]. All studies reported BOP, suppuration on probing, PPD, and PD as the primary outcomes. Clinical and microbiological measurements were performed in four studies [28,36,37,38]. Participants were recruited from departments of periodontology [37,38] and dental school [28,36]. One study did not specify a setting [39].

The five studies included a total of 250 patients and 333 implants. Four studies compared the efficacy of local/topical antibiotics with mechanical debridement or scaling and root planning without surgical treatment [28,36,37,39], while one study used a surgical approach [38]. Two studies compared minocycline microspheres with 0.1 mL chlorhexidine gel 1% [28,36], one study compared minocycline ointment with placebo ointment [38], while the remaining two studies compared, respectively, doxycycline and minocycline, this last used alone or with metronidazole, without adding additional treatments in the control group [37,39].

### 3.4. Doxycycline Efficacy

Only one study investigated the effectiveness of the local application of doxycycline related to usual treatment: Butcher et al. compared the effect of 8.5% doxycycline and no additional treatment in a sample of 28 patients and 48 implants (treatment group: 14 patients; control group: 14 patients). After executing removal prosthetic restoration, abutment sterilisation, irrigation with 0.2% of chlorhexidine, and implant scaling, local debridement together with 8.5% doxycycline hyclate (treatment group) was compared with local subgingival debridement. After four months, statistically significant differences between groups emerged for the attachment level changes, BOP and PD, showing a more substantial improvement in the group treated with doxycycline than in the control group (*p* < 0.05) [39].

### 3.5. Minocycline Efficacy

Four studies analysed the effectiveness of minocycline in reducing peri-implantitis signs. Two studies (*n* = 62 patients, 125 implants, treatment group: 33 patients/73 implants; control group: 29 patients/52 implants) compared the submucosal administration of minocycline microspheres and 0.1 mL chlorhexidine gel 1%. Statistically significant improvements in all clinical and microbiological outcomes within both groups (*p* < 0.001) after 12 months emerged. Nevertheless, both studies failed to observe statistically significant differences between groups at the end of the planned follow-up period [28,36]. Furthermore, no significant differences emerged between the two groups in the mean total number of bacteria in both studies.

In 2019, Cha et al. investigated the effectiveness of 10 mg of local minocycline in 0.5 g of ointment in a sample of 46 patients/46 implants, comparing results with those observed in a placebo group, treated with a placebo ointment, and using the same modality of administration already used in the treatment group. In both groups, surgical treatment was performed. After 6 months, at the deepest site, a statistically significant improvement emerged only for the gingival index (treatment: 0.96 ± 0.86, control: 0.41 ± 0.85, *p* = 0.035), while the mean at four sites showed a statistically significant difference between groups for PPD (treatment: 2.68 ± 1.73; control: 1.55 ± 1.86, *p* = 0.039) and GI (treatment: 0.83 ± 0.60; control: 0.40 ± 0.68, *p* = 0.026). The number of red-complex bacteria decreased in both groups [38].

Recently, Park et al., in a randomised double-blind three-arm clinical trial, compared the effectiveness of mechanical debridement (identified as group NST) alone, mechanical debridement combined with minocycline ointment (identified as group MC), or combined with minocycline–metrodinazole ointment (identified as group MM). After 4 months, statistically significant differences emerged between the MM group and the NTS group for PPD (MM: −2.71 ± 1.90; NTS: −2.03 ± 1.38, *p* = 0.0023) and BOP (MM: −0.66 ± 0.53; NTS: −0.38 ± 0.49, *p* = 0.0381). Bacteria counts of *P. gingivalis*, *T. forsythia*, *T. denticola*, *P. intermedia*, *C. rectus*, and *F. nucleatum* showed a significant decrease in the MM and MC groups. In the NTS group, a significant decrease was observed only for *P. gingivalis* [37].

## 4. Discussion

The pathophysiology underlying the development of peri-implantitis involves pathogenic microorganisms. Therefore, the proposed treatment modality should minimise the microbial load, decontaminate the surface of the implant, and eliminate peri-implant mucosal inflammation. This approach would be critical in preserving the bone structure supporting the implant and initiating the regeneration of the bone lost during the disease process.

Although mechanical debridement and oral health instructions have been demonstrated to effectively minimise inflammatory signs and symptoms, current findings can be considered inconclusive. On the one hand, many studies have employed antiseptics, systemic or local antimicrobial therapies, and regenerative therapies as adjunctive to mechanical decontamination; on the other hand, in non-surgical peri-implantitis treatment, the use of mechanical debridement alone could be ineffective [8,40]. Therefore, non-surgical approaches should be limited to peri-implant mucositis management as, in cases of bone loss characteristic of peri-implantitis, the therapy fails to resolve lesions secondary to the inflammatory process.

Therefore, surgery is recommended to allow direct decontamination of the implant surface and complete granulation tissue removal [40]. However, non-univocal findings have emerged: success rates of surgical treatments vary among studies from 79%, with a decrease to 63% after 5 years [41], to 23% [42]. Chemical adjuvants and mechanical debridement followed by systemic antibiotics are adjunctive to the surgical approaches to enhance therapeutic effects. In this case, the use of different chemical adjunctive peri-implantitis treatments failed to establish a single recommended protocol [8,40,43].

Therefore, determining new non-surgical and surgical approaches using local/topical antibiotics may represent a fundamental approach to control peri-implantitis.

The use of local/topical antibiotics as an adjunctive therapy of mechanical debridement has reported favourable but mild evidence. Therefore, we had hypothesised that local/topical antibiotics could significantly improve clinical peri-implant disease compared to mechanical debridement or scaling root planning and could enhance the efficacy of the surgical approach. Antimicrobial agents applied locally during periodontal surgical treatment may improve the outcomes and minimise the manifestations of peri-implantitis [44]. To focus better on the topic of this systematic review, only RCT studies, without regard to surgical or non-surgical approach, and which compared local antibiotic application with mechanical debridement, scaling and root planning, or placebo ointment, were included to reduce the overestimation of the actual local/topical antibiotics effectiveness. The findings of this systematic review failed to demonstrate the superiority of local/topical antibiotics.

Minocycline reduces inflammatory cytokines, producing significant decreases in PD and BOP, although repeated applications are needed to maintain the therapeutic results [28,36,37,38], increasing the risk for bacterial resistance [45]. When used in combination with metronidazole, minocycline showed efficient antimicrobial action [37]. Combining the evidence of different included studies about the efficacy of minocycline, we hypothesised that these results should not be considered the direct effect of minocycline but the effect of metronidazole, which had already shown a positive antimicrobial effect against a wide range of microorganisms [46].

Minocycline added as an ointment in the surgical approach did not show significant improvements compared to placebo ointment [38]: although a strong and significant reduction was noted within the treatment group, the lack of a statistically significant difference between treatment and placebo ointment applications should be explained as the result of an ineffective action of antibiotic treatment.

The topical application of tetracycline has been reported to produce remarkably positive effects on clinical parameters such as CAL, sulcular bleeding index (SBI), and PD [47]. Furthermore, using local/topical antibiotics at the surgical site could decrease the inflammation following the placement of an implant [48].

Mombelli et al. [30] reported improvements in the clinical and microbiological parameters in the therapeutic management of peri-implantitis using tetracycline applied topically. Erythromycin eye ointment and minocycline ointment were administered at the surgical implant site topically. Both ointments produced comparable results regarding the healing process, particularly in the early stage [48].

Only one study analysed the effect of doxycycline in reducing peri-implantitis symptoms [39]: the findings of this included study appeared encouraging. Doxyclycline is a second-generation semisynthetic derivative of tetracycline, with efficacious antimicrobial action [46] in periodontitis therapy [49]. Similarly to minocycline, doxycycline effectively reduces the depth of pockets and has strong antimicrobial activity against *Prevotella intermedia/nigrescens*, *Fusobacterium* sp., *Bacteroides forsythus*, and *Campylobacter rectus* [30].

An innovative gel composed of doxycycline and metronidazole compared to CBB and planktonic species showed promising results in an in vitro study. For 13 days, the positive effect continued, and the authors reported that the application of the new gel could be discussed in cases of peri-implantitis [50]. Furthermore, Madi et al. (2018) demonstrated that nano-doxycycline gel combined with scaling and root planning produced significant anti-inflammatory effects [51].

Then, despite marked improvements in all peri-implantitis symptoms (e.g., BOP, PPD, GI, and PI) in the group treated with L/T antibiotics, the evidence failed to reveal any remarkable differences between the control and test groups for all microbiological and clinical parameters.

Comparing the findings of this review with those observed in observational studies, many contrasting results emerged too. Schenk et al. and Hallstrom et al., administering, respectively, 43% tetracycline fibres as adjunctive therapy to mechanical debridement for ten days and mechanical debridement (rubber polishing + titanium curettes) + oral health instructions + systemic azithromycin (4 days), failed to demonstrate any significant difference between the treatment and control group without local antibiotic treatment [52,53]. Contrarily, Schwartz et al. reported a considerable improvement in clinical and radiographic parameters when combining non-surgical peri-implantitis treatment with chlorhexidine irrigation and minocycline applied locally [54]. Promising results were also confirmed by Heo et al., who demonstrated that the use of a simple non-surgical approach combining intrasulcular chlorhexidine and the local delivery of minocycline improved clinical and radiographic parameters [54]. According to Heitz-Mayfield, significant improvements in clinical and microbiologic parameters were achieved at 3 months in treating peri-implantitis using the local application of minocycline microspheres as part of the CIST protocol [55]. From our findings, local antibiotic applications could reduce PD, but their efficacy appears limited to a short period.

In some case series and case reports, many encouraging results also emerged. Mensi et al. reported improvements in PPD and BOP using a Multiple Anti-Infective Non-Surgical Treatment (MAINST) protocol that consisted of two treatments, each executed in two consecutive weeks, based on air polishing, ultrasonic, manual debridement, chlorhexidine rinse, and a final pocket filling with doxycycline gel 14% (Ligosan^®^ Kulzer Gmbh, Hanau, Germany) [56]. The same results were also reported in Moura et al. and Diachova et al., where improvements in PD [57,58] and BOP [58] were registered after using local doxycycline. These findings confirmed Butcher et al. (2004), who demonstrated a significant improvement in peri-implant disease after applying doxycycline as a local antibiotic [39].

The contrasting findings that emerged could be explained by the difficulty of local/topical antibiotics to arrive at a high concentration on the implant surface. This difficulty could discourage clinicians from adopting local/topical antibiotics. To achieve a high concentration of local/topical antibiotics, clinicians are required to increase the total amount of local/topical antibiotics, consequently increasing the risk of antimicrobial resistance and adverse effects [20].

The synthesis of the current data indicated that peri-implant mucositis non-surgical treatment, concerning PD and BOP scores, does not favour local/topical antibiotics or local antiseptic treatment as a combination with mechanical detoxification alone [59,60]. These findings were confirmed in a recent systematic review, which also demonstrated that the adjunctive treatment fails to facilitate the effectiveness of plaque removal in reducing PD (such as air abrasive device, systemic and local/topical antibiotics) and BOP (such as septic or systemic/local antibiotics) scores at the sites with peri-implant mucositis [5]. Contrarily, for non-surgical peri-implantitis treatment, the BOP scores favoured either local antibiotics [28,30,36,39,45,56,61,62,63,64] adjunctive therapy or alternative plaque control measures [63] over respective control therapeutic managements. Therefore, from the above evidence, local/topical antibiotics as an adjunctive treatment are more effective in peri-implantitis than peri-implant mucositis.

Regarding the microbiological outcome, all included studies demonstrated a reduction in bacteria, although without statistically significant differences between treatment and control groups. Only Park et al. showed the more robust activity of local minocycline in reducing *P. gingivalis*, *T. forsythia*, *T. denticola*, *P. intermedia*, *C. rectus*, and *F. nucleatum* after 12 weeks of treatment. This result is encouraging, but it is not enough: contaminated implant surfaces represent a reservoir of peri pathogens in both healthy and diseased implants, as a result of the “circular model” of bacterial contamination [65,66].

Comparing our findings with the current literature, moderately significant results were demonstrated in a recent systematic review and meta-analysis that investigated the efficacy of local antibiotic administration in reducing PPD and BOP using a different approach than ours [67]. After the screening of an initial 95 articles and including different study designs (i.e., RCTs, case series, cohort, and case–control studies) and different control groups (e.g., photochemotherapy), the authors concluded that local antibiotics reduce PPD and BOP in comparison to the control group without local antibiotics, but a high level of heterogeneity was highlighted as a study limitation that could have reduced the quality of the encountered evidence [67].

A high level of heterogeneity was observed among the included studies, mainly due to the surgical or non-surgical approach, materials employed, operators, local antibiotic dosage and delivery, different control groups, study protocol, and microbiological outcomes. These factors reduced the quality of the evidence. Even in those studies which used the same local antibiotics, i.e., minocycline, differences in follow-up hampered a valid comparison [28,36,37,38]. None of the included studies reported adverse effects after local antibiotic administration. The incidence of side effects should be verified in future studies. To date, we know that adverse effects were demonstrated after systemic antibiotic administration in periodontitis (e.g., gastrointestinal disorders, allergic reaction, headache, or fever) [68].

The limitations of this review could be addressed as follows. First, only five RCTs were eligible for this review and the risk of bias was not assessed because only two of the included were almost recent and therefore conducted according to the CONSORT guidelines. Secondly, the multiplicity of antibiotics prevents us from determining an actual clinical effect, given a different host response. Finally, diverse follow-up periods, implant types, locations, outcomes, and diagnostic criteria determined high heterogeneity among studies, which prevents definitive conclusions from being made.

Future clinical trials are suggested in order to compare the effect of different local/topical antibiotics in the presence of diverse microbiological factors and immunological parameters. Moreover, accurate comparisons of the antibiotics’ impact with similar biological activity profiles could provide a validated and conclusive protocol.

## 5. Conclusions

In conclusion, the extensive use of implants raises the question of future peri-implant diseases. Unfortunately, there are very few existing studies that report on etiological peri-implantitis treatment in isolation. However, enough current studies have proven that in peri-implantitis treatment, the elimination of the bacterial biofilm from the implant surface either through surgical or non-surgical access should be supplemented by chemical adjunct therapeutic approaches through surgical access. Unfortunately, to date, few existing studies also compare the efficiency of different types of chemical treatments as adjuncts to peri-implantitis management. Therefore, it is impossible to establish a firm recommendation of the chemical adjunct that should be used for peri-implantitis management. To the best of our knowledge, this work is the first literature review focused on local/topical antibiotics.

However, the administration of local/topical antibiotics has been shown to have positive clinical outcomes in peri-implantitis. Nevertheless, a protocol with strong scientific evidence cannot be derived from the studies conducted to date. Therefore, more studies with homogeneous designs and longer follow-up are desirable considering the lack of a definable gold-standard treatment to date.

## Figures and Tables

**Table 1:** Included studies.

Author	Country	Number Pz and Imp	Setting	Clinical Characteristics	Surgical/Non-Surgical	Treatment	TG	CG	FU (Months)	Main Findings
Buchter et al. (2004) [39]	Germany	28 pz 48 imp TG: 14 pz CG: 14 pz	NR	Chronic peri-implantitis PD > 5 mm; bone defects exceeding 50% of the length of the implant on radiographs	Non-surgical	Removal prosthetic restoration + abutment sterilised + 0.2% CHX irrigation + implant scaling	8.5% DOX	No additional treatment	4	TG: CAL: 5.32 ± 0.33, 4.17 ± 0.30 (*p* < 0.001); PD: 5.64 ± 0.32, 4.49 ± 0.29 (*p* < 0.001); BOP: 0.54 ± 0.07, 0.27 ± 0.06 (*p* = 0.001) CG: CAL: 5.51 ± 0.27, 5.18 ± 0.33 (*p* < 0.001); PD: 5.68 ± 0.28, 5.4 ± 0.34 (*p* < 0.001); BOP: 0.63 ± 0.06, 0.50 ± 0.07 (*p* = 0.001) Difference between groups: CAL: *p* = 0.024, PD: *p* = 0.046, BOP: *p* = 0.01
Renvert et al. (2006) [36]	Sweden	30 pz 30 imp TG: 16 pz CG: 14 pz	Kristianstad University	BOP or pus + PD ≥ 4 mm + presence of putative pathogenic bacteria + loss of bone ≤ 3 threads on radiographs	Non-surgical	OHI + supra and sub mucosa scaling + rubber cup polish	Submucosal administration of MIN microspheres	Submucosal administration of 0.1 mL CHX gel 1%	12	TG: PI (%): 50 ± 25, 27 ± 24; BOP/BOS (%): 88 ± 12, 71 ± 22 PD (mm) 3.9 ± 0.7, 3.6 ± 0.6 CG: PI (%): 60 ± 49, 27 ± 45; BOP/BOS (%): 89.2 ± 17.2, 63.5 ± 19.1; PD (mm) 3.87 ± 1.16, 3.72 ± 1.02. Comparison between groups at six months: PI (*p* > 0.05), BOP/SOP (*p* ≤ 0.01 all four sites/implants, *p* > 0.05 deepest site/implant), PD (*p* ≤ 0.001). Comparisons between groups at 12 months: PI (*p* > 0.05), BOP/SOP (*p* > 0.05 all four sites/implants, *p* ≤ 0.05 deepest site/implant), PD (*p* ≤ 0.001 all four site/implants, *p* ≤ 0.01 deepest site/implant). No statistical significance emerged between the two antimicrobials for any bacteria at any time point.
Renvert et al. (2008) [28]	Sweden	32 pz 95 imp TG: 17 pz/57 imp CG: 15 pz/38 imp	Kristianstad University	PD ≥ 4 mm + BOP and/pr pus + radiographic bone loss ≤ 3 threads + occurrence of anaerobic bacteria + presence of one or more bacteria	Non-surgical	NR	MIN microspheres	0.1 mL 1% CHX gel	12	Treatment group: LPS (%): 50 ± 50, 22 ± 42; BOP/BOS (%): 86.5 ± 20.1, 48.1 ± 20.7; PD (mm) 3.85 ± 1.04, 3.55 ± 0.98; Control group: LPS (%): 60 ± 49, 27 ± 45; BOP/BOS (%): 89.2 ± 17.2, 63.5 ± 19.1; PD (mm) 3.87 ± 1.16, 3.72 ± 1.02. Comparison between groups at six months: LPS (*p* = 0.003); PD (*p* > 0.05), BOP/SOP (*p* < 0.001). Comparisons between groups at 12 months: LPS (*p* > 0.05); PD (*p* > 0.05), BOP/SOP (*p* < 0.001). No statistically significant differences in the mean total numbers of bacteria between and within groups after 12 months.
Cha et al. (2019) [38]	South Korea	46 pz 46 imp TG: 24 pz CG: 22 pz	Department of Periodontology	Peri-implant bone loss > 2 mm; PPD > 5 mm; concomitant BOP	Surgical treatment	Surgical treatment	Local MIN (10 mg of minocycline in 0.5 g of OIN)	PBO OIN	6	PPD: TG: 3.58 ± 2.32; CG: 2.45 ± 2.13; *p* = 0.094. BOP: TG: 0.58 ± 0.50; CG: 0.32 ± 0.57; *p* = 0.102. PI: TG: 0.12 ± 0.90; CG: 0.23 ± 1.07; *p* = 0.728. GI: TG: 0.96 ± 0.86; CG: 0.41 ± 0.85; *p* = 0.035. Number of red-complex bacteria decreased in both groups, without statistically significant differences between groups.
Park et al. (2021) [37]	Korea	114 pz 114 imp MM: 38 pz MC: 39 pz NST: 37 pz	Department of Periodontology	Implant inserted at least 1 year previously; PPD ≥ 5 mm; presence of BOP, SoP, and peri-implant bone loss in a periapical radiograph	Non-surgical	OH + non-surgical debridement	MD + MTZ-MIN OIN (MM); MD + MIN OIN (MC)	MD	4	MM: PPD −2.71 ± 1.90; BOP: −0.66 ± 0.53; Pus Suppuration: −0.18 ± 0.39; PI: −0.71 ± 0.80. MC: PPD −2.51 ± 1.82; BOP: −0.59 ± 0.50; Pus Suppuration: −0.31 ± 0.46; PI: −0.54 ± 0.76. NTS: PPD −2.03 ± 1.38; BOP: −0.38 ± 0.49; Pus Suppuration: −0.32 ± 0.53; PI: −0.35 ± 0.89. Statistically significant differences were found for PPD and BOP between MM and NTS (*p* = 0.0023, *p* = 0.0381). After 12 weeks, significant decreases in the counts of *P. gingivalis*, *T. forsythia*, *T. denticola*, *P. intermedia*, *C. rectus*, and *F. nucleatum* in MM and MC group. In the NST group, decrease only for *P. gingivalis*

Legend: BOP: bleeding on probing; CAL: clinical attachment level; CG: control group; CHX: chlorhexidine; DOX: doxycycline; FU: follow-up; imp: implants; MC: mechanical debridement + minocycline ointment; MD: mechanical debridement; MIN: minocycline; MM: mechanical debridement + minocycline–metronidazole ointment; MTZ: metronidazole; OH: oral hygiene; OHI: oral hygiene instructions; OIN: ointment; PBO: placebo; PD: pocket depth; PI: plaque index; PPD: probing pocket depth; SOP: suppuration on probing; TG: treatment group; pz: patients; RCT: randomised clinical trial.

## Data Availability

The data presented in this study are available on request from the corresponding author.
